# Development and external validation of the ‘Flower-FFQ’: a FFQ designed for the Lifelines Cohort Study

**DOI:** 10.1017/S1368980021002111

**Published:** 2022-02

**Authors:** Elske M Brouwer-Brolsma, Corine Perenboom, Diewertje Sluik, Anne van de Wiel, Anouk Geelen, Edith JM Feskens, Jeanne HM de Vries

**Affiliations:** Division of Human Nutrition and Health, Wageningen University, PO Box 17, 6700 AA Wageningen, The Netherlands

**Keywords:** Dietary assessment, FFQ, Validation

## Abstract

**Objective:**

FFQ assess habitual dietary intake and are relatively inexpensive to process, but may take up to 60 min to complete. This article describes the validation of the Flower-FFQ, which consists of four short FFQ measuring the intake of energy and macronutrients or specific (micro)nutrients/foods that can be merged into one complete daily assessment using predefined algorithms.

**Design:**

Participants completed the Flower-FFQ and validated regular-FFQ (*n* 401). Urinary N (*n* 242) and K excretions (*n* 361) were measured. We evaluated: (1) group-level bias, (2) correlations and (3) cross-classification.

**Setting:**

Observational study.

**Participants:**

Dutch adults, 54 ± 11 (mean ± SD) years.

**Results:**

Flower-FFQ1, Flower-FFQ2, Flower-FFQ3 and Flower-FFQ4 were completed in ±24, 9, 8 and 9 min (±50 min total), respectively. The regular-FFQ was completed in ±43 min. Mean energy (flower *v*. regular: 7953 *v*. 8718 kJ/d) and macronutrient intakes (carbohydrates: 204 *v*. 222 g/d; protein: 75 *v*. 76 g/d; fat: 74 *v*. 83 g/d; ethanol: 8 *v*. 12 g/d) were comparatively similar. Spearman correlations between Flower-FFQ and regular-FFQ ranged from 0·60 to 0·80 for macronutrients and from 0·40 to 0·80 for micronutrients and foods. For all micronutrients and foods, ≥ 78 % of the participants classified in the same/adjacent quartile. The Flower-FFQ underestimated urinary N and K excretions by 24 and 18 %; 75 and 73 % of the participants ranked in the same/adjacent quartile.

**Conclusion:**

Completing the Flower-FFQ required 50 min with a maximum of 25 min per short FFQ. The Flower-FFQ has a moderate to good ranking ability for most nutrients and foods and performs sufficiently to study diet–disease associations.

Prospective cohort studies provide the unique opportunity to characterise potential risk factors before disease onset^(1)^ and are therefore very suitable to investigate potential diet–disease associations^(2)^. In many well-known cohort studies, the food FFQ has been the method of choice to assess dietary intake^(3–5)^. FFQ capture individual habitual long-term dietary intake and are relatively easy and inexpensive to process. However, FFQ may also be time-consuming to develop and complete^(6)^. To illustrate, an extensive 200-item FFQ addressing the intake of energy, macronutrients and the majority of micronutrients is usually completed in approximately 45–60 min. This completion time is considered burdensome by many respondents and often results in the return of incomplete questionnaires. Additionally, questions at the end of a questionnaire are also more likely to be affected by measurement error compared with questions in the beginning of a questionnaire^(7)^. To reduce participant burden and associated measurement error, we decided to develop a new type of FFQ for the Lifelines Cohort Study, a multi-disciplinary prospective population-based cohort study including over 160 000 Dutch citizens^(8)^.

This new-FFQ, called Flower-FFQ, was designed to derive a valid long-term estimate of the habitual dietary intake using an innovative approach that combines one main questionnaire (representing the heart of the flower) with three short complementary questionnaires (representing the petals) administered at different time points. Each questionnaire focusses on different nutrients and/or foods (Fig. 1). Within the Lifelines Cohort Study, the four questionnaires were sent to the participants over a period of 5 years, assuming stable food consumption patterns over time. Food item selection for the Flower-FFQ was based on a standardised approach, that is, for each food the contribution to the absolute intake and the between-person variability of the selected nutrient was calculated where the Dutch National Food Consumption Survey (DNFCS) served as the reference. Foods contributing to at least 80 % cumulative contribution of absolute intake and/or explaining at least 80 % of the between-person variability were included in the Flower-FFQ^(9,10)^. Based on the number of items queried in the Flower-FFQ, the estimated time needed to complete all four FFQ would be approximately 60–75 min. The additional time needed to complete the Flower-FFQ compared with a ‘regular’ FFQ relates to the fact that the four Flower-FFQ contain overlapping items, which is crucial to ensure proper linkage. Therefore, we assumed that the time needed to complete the Flower-FFQ would be about 20–25 % more than the time needed to complete a comparable regular-FFQ, but that the administration mode (i.e., four short FFQ) would be more convenient for the participant and less sensitive to errors.

**Fig. 1:**
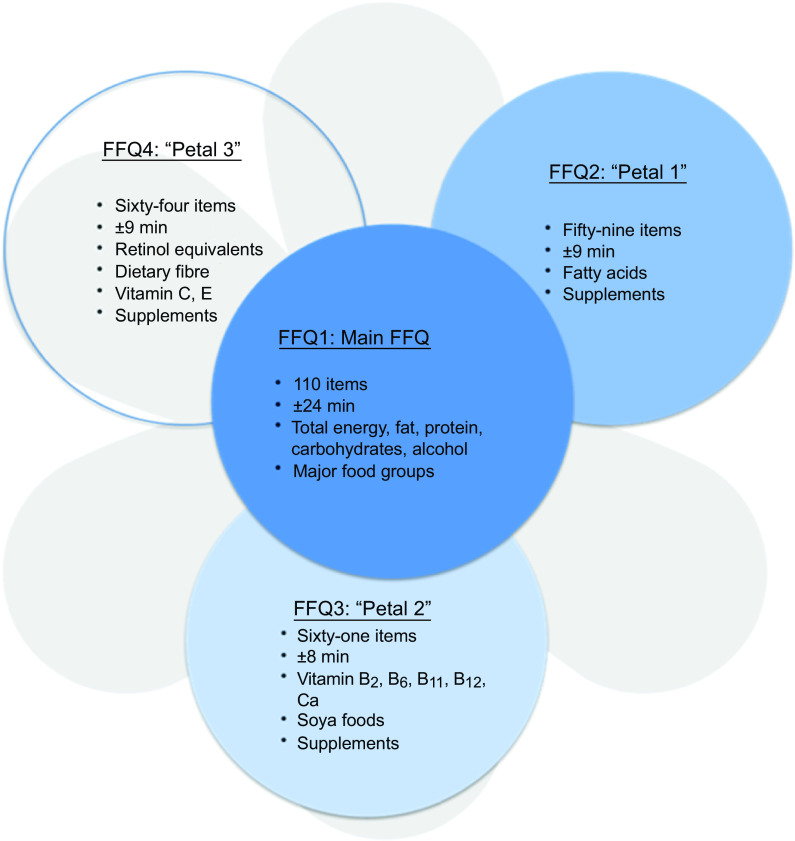
The Flower-FFQ constituted of the main FFQ and three complementary ‘Petals’: each petal indicates the number of items per short-FFQ, estimated completion time and assessed nutrients, food groups and/or supplements

Validation of this newly developed Flower-FFQ is essential to show to what extent measurement error may interfere with diet–disease relationships observed in future studies that use this FFQ. The relative validity can be examined by comparing the FFQ with a reference method. Usually this comparison is made by exploring correlations between the new FFQ and the reference method^(6)^, which provides an impression of the strength and direction of association^(11)^. Ideally, these correlation coefficients are supported by other validity measures, such as cross-classification data showing whether or not participants are classified in the same category with the two methods (i.e., also ranking ability), and *t* tests or group-level bias (e.g., when absolute intakes are important)^(6,11)^. To evaluate actual validity, the use of biomarkers is essential. However, to date there are still just a few validated nutritional recovery biomarkers, which include urinary N, K, Na and doubly labelled water to estimate absolute intakes of protein, K, Na and energy, respectively^(12)^.

Within the Lifelines Cohort Study, no other dietary assessment method than the Flower-FFQ has been administered and as such no reference method is available to quantify the habitual dietary intake within that cohort. Therefore, an external validation study on the Flower-FFQ was conducted within the Nutrition Questionnaires plus study^(10,13)^. Participants of the Nutrition Questionnaires plus study completed a Flower-FFQ, a validated regular-FFQ^(14,15)^ and provided urine to determine urinary N and K excretions as commonly accepted recovery markers for the intake of protein and K^(12)^. This article describes the development of the Flower-FFQ for the Lifelines Cohort Study and its external validation within the Nutrition Questionnaires plus study.

## Methods

### Participants

Between June 2011 and February 2013, 2048 Dutch men and women aged 20–70 years were enrolled in the National Dietary Assessment Reference Database^(10)^ and the Nutrition Questionnaires plus study^(13)^. Participants were recruited in the surroundings of Wageningen, the Netherlands. Participants were eligible for participation in the study when they were between 20 and 70 years of age at the time of recruitment, competent to make own decisions and provided a written informed consent. Participants were not eligible when they were unable or unwilling to comply with the study procedures, enrolled in another study in same period or not able to read and speak Dutch. All participants gave written informed consent before commencement of the study.

### Population for analyses

The current analyses were conducted using data of participants with complete dietary data – including data obtained with the Flower-FFQ and regular-FFQ (*n* 404). Participants with unreliable or incomplete Flower-FFQ and/or regular-FFQ data (i.e., men with energy intakes <3347 kJ (<800 kcal) or >17 573 kJ (>4200 kcal), women <2092 kJ (<500 kcal) or >14 644 kJ (>3500 kcal))^(16)^ were excluded (*n* 3) and as such 401 participants were included in the analyses. All participants gave written informed consent. Analyses on protein intake and urinary N excretion could be performed in a subsample of 242 participants; 361 participants provided data on K intake and urinary K excretion. The study was approved by the ethical committee and was conducted according to the declaration of Helsinki.

### Flower-FFQ

**Fig. 2:**
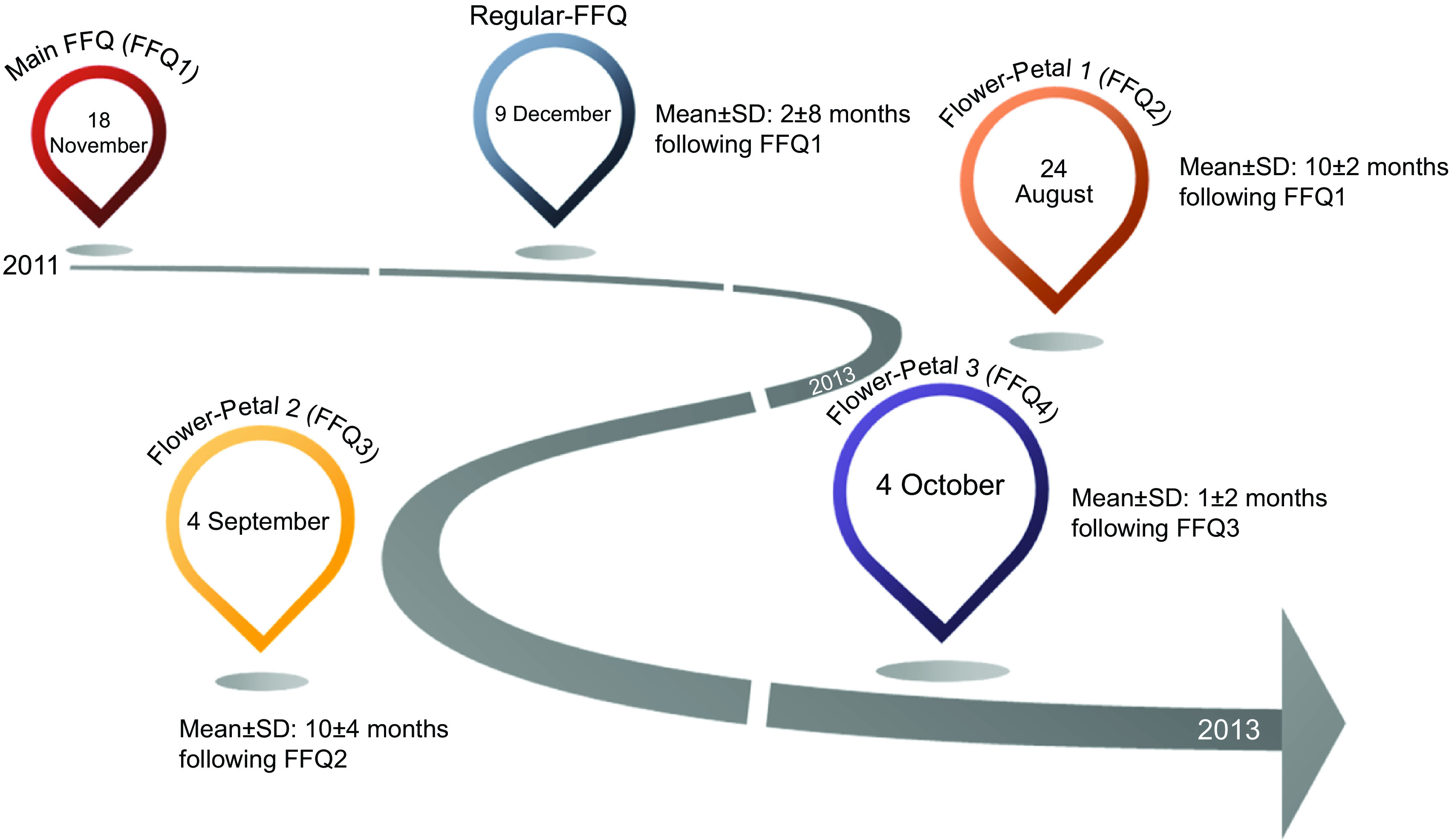
Timings of the measurements of the Flower-FFQ validation study

The name Flower-FFQ is derived from its design. The FFQ consists of one main FFQ (FFQ1), which symbolises the heart of the flower and measures the intake of energy and macronutrients. The three complementary FFQ symbolise the flower petals and focus on specific (micro)nutrients and food components, that is, the fatty acids FFQ providing information on SFA, MUFA, PUFA, EPA and DHA (focussing on, e.g., meat, fish, fats and oils) (FFQ2); the B-vitamins FFQ providing information on vitamin B_2_, vitamin B_6_, folic acid, vitamin B_12_ and Ca (focussing on, e.g., dairy products, meat products, vegetables and fruit) (FFQ3) and the vitamin ACE FFQ providing information on retinol equivalents, vitamin C, vitamin E and dietary fibre (focussing on, e.g., vegetables, fruits, bread, grains (including pasta and rice), and fats and oils) (FFQ4). Figure 1 graphically displays the Flower-FFQ, its design aspects and nutrients of focus. The timing of the four FFQ is displayed in Fig. 2. Food lists were compiled using the Dutch FFQTOOL™ by selecting the food items with the highest absolute contribution to the selected nutrient intakes, which was based on the DNFCS of 1998^(17)^. Specifically, for each food the contribution to the absolute intake and the between-person variability of the selected nutrient was calculated. Foods contributing to at least 80 % cumulative contribution of absolute intake or explaining at least 80 % of the between-person variability were included in the Flower-FFQ. Combined, the four FFQ cover 212 items and ≥92 % of the absolute level of intake and ≥90 % of the between-person variability of each nutrient as assessed by 2-d food records in the DNFCS 1998^(17)^. Questions pertaining to frequency were completed by selecting answers ranging from ‘never’ to ‘6–7 d per week’. Portion sizes were estimated using natural portions and commonly used household measures. Average daily energy and nutrient intakes were calculated by multiplying consumption frequency by portion size and nutrient content per gram, as indicated in the Dutch food composition table of 2011^(18)^. In general, the main FFQ assessed the frequency and number of servings of all major food groups according to the Dutch food composition table, and the three complementary FFQ assessed the frequency of the food subgroups. The intake of specific nutrients or food subgroups as assessed by the complementary FFQ was calculated by combining the number of servings of the major food reported in the main FFQ with the specific type reported in the complementary FFQ. For instance, the main FFQ assessed the consumption frequency and number of servings of rice, and the complementary FFQ identified the type of rice, that is, white or brown. Prior to data collection, we decided that in case of inconsistencies between the main FFQ and complementary FFQ, the data of the main FFQ would be considered superior, which were amongst others based on the theory that question ordering can impact retrieval when asking about a series of events that occur over time, for example, remembering one event may help to remember the next (or previous) event in the sequence^(19)^. As the main FFQ registered the overall habitual diet, without many details, we felt that this FFQ was the most efficient one to help participants remember their food intake. To illustrate, if the main FFQ indicated the consumption of a food while it was not reported in the complementary FFQ, the particular food subgroups received a weighted consumption average. If the main FFQ indicated that a food was not consumed while it was consumed according to a complementary FFQ, the food was recorded as not consumed. However, data checks eventually showed that inconsistencies between the main FFQ and petal FFQ appeared to be negligible. To illustrate, in the Flower-FFQ vegetables were covered by nineteen items, and as such we assumed that this food group was at a relatively high risk of being affected by inconsistencies between the main and petal FFQ. Despite that, we only identified five participants reporting a ‘zero-intake’ in the main FFQ, while they did report vegetables in the petal FFQ. However, in the petal FFQ the reported vegetables were raw vegetables, which were intentionally not asked in the main FFQ. As such, the item on raw vegetables was completely calculated based on the petal data (including the information on grams), and thus no inconsistencies were observed for vegetable intake. Subsequently, we did a similar analysis for the food group bread, which was covered by eleven items. Data of the main FFQ showed three ‘zero-intake’ reporters, of whom none reported bread consumption in the petal FFQ. Finally, we explored potential inconsistencies for rice and pasta; these foods are usually consumed on 1–2 d per week as part of the Dutch diet. Rice and pasta were both covered by two items that distinguished between whole wheat and plain types. For pasta, twenty-six ‘zero-intake’ reporters were identified and for rice there were sixty-eight ‘zero-intake’ reporters; none of them reported either pasta or rice in the petal FFQ. The Flower-FFQ was administered online, randomly distributed over the week, via the open-source survey tool LimeSurvey^TM^ (LimeSurvey Project Team/Carsten Schmitz.) within a period of 2 years, assuming stable food consumption patterns over time. This assumption is supported by stable BMI measures over the course of the current study (i.e., at baseline, year 1 and year 2 (mean ± sd) 25·6± (3·7) (*n* 401), 25·4± (3·6) (*n* 399) and 25·6± (7·5) (*n* 301), respectively). The main FFQ was completed ±5 months following baseline, for example, participants included in June 2011 completed the main FFQ in November 2011. About 10 months later (Augusts 2012), these participants completed petal 1, followed by petal 2 another year later (September 2013). Finally, petal 3 was completed 1 month after the completion of petal 2 (October 2013). A sample of the Flower-FFQ (in Dutch) can be obtained by contacting the authors.

### Food frequency questionnaire

Habitual dietary intake was also assessed by a validated semi-quantitative regular-FFQ including 183 items, where the reference period of the FFQ was the previous month. Previous validation studies of this FFQ showed acceptable to good correlation for the intake of energy (*r* 0·65 with phone-based 24-h dietary recalls), fats (*r* ranges between 0·24 and 0·33 for adipose tissue), dietary fibre and a selected number of vitamins and food groups (*r* 0·82 with phone-based 24-h dietary recalls)^(14,15,20)^. This FFQ covered ≥ 96 % of the absolute level of intake and ≥ 95 % of the between-person variability of each nutrient as assessed in the DNFCS of 2011^(17)^. Questions relating to consumption frequency were followed by answer categories ranging from ‘never’ to ‘6–7 d per week’. Portion sizes were estimated using natural portion sizes and commonly used household measures. Subsequently, energy and nutrient intakes were calculated through multiplying the consumption frequency by portion size and nutrient content (grams) as indicated in the Dutch food composition table of 2011^(18)^. The FFQ was administered online, randomly distributed over the week, via the open-source survey tool LimeSurvey^TM^; the first participants completed this regular in December of 2011, about 1 month following the main FFQ.

### Urine sampling

In this validation study, data of a single 24-h urine collection were used in order to determine urinary N and K excretions as commonly accepted recovery markers for the intake of protein and K^(12)^. Urine was collected at baseline and started with the second voiding after waking up and finished after the first voiding after waking up the next day. Urine collections were handed in at the hospital and transported to the study centre, where they were mixed, weighed, aliquoted and stored at –20°C until further analysis. Participants received three 80 mg para-aminobenzoic acid tablets to check for completeness of the urine collections. Total 24-h N excretion was determined by the Kjeldahl technique (Foss KjeltecTM 2300 analyser)^(21)^. Urinary protein was calculated with the following formula: 6·25 × (urinary N/0·81), accounting for approximately 19 % faecal and skin losses^(22)^. Urinary K was measured with an ion-selective electrode on a Roche 917 analyser, assuming a urinary excretion of 81 % for K^(23)^. As the Observing Protein and Energy Nutrition study did not observe an effect of the exclusion of participants with incomplete urines on correlations and attenuation factors^(24)^, our primary analyses on protein and K were conducted using the data from all urine samples. Secondary analyses confirmed that also within our sample excluding those with a para-aminobenzoic acid recovery < 85 % did not substantially alter the results.

### Additional measurements

Health and lifestyle questionnaires were completed at baseline via the online open-source survey tool LimeSurvey^TM^. Questionnaires included items on demographics, educational attainment and smoking habits^(10,13)^. Physical examinations were also conducted at baseline at the study centre according to a standardised protocol by a well-trained staff. Height was measured with a stadiometer (SECA) to the nearest 0·1 cm, without shoes. Weight was measured on a digital scale (SECA) to the nearest 0·1 kg, without shoes and sweaters and empty pockets. BMI was calculated as weight/heigth^2^.

### Statistical analysis

Participant characteristics are reported as mean with SD (mean ± sd) or *n* with percentages (*n* (%)). Means with sd are also provided for intakes of energy, macronutrients and food groups. Macronutrients and ethanol were additionally expressed in energy densities to adjust for energy. Although the main focus of this validation study was on the ranking ability of the Flower-FFQ, absolute intake differences between the Flower-FFQ and regular-FFQ were expressed as group-level bias (i.e., a measure of misreporting): (mean intake Flower-FFQ/mean intake reference method) × 100 – 100. For the intake of protein and K, the level of bias was evaluated by plotting the distribution of the self-reported intake against the distribution of the intake based on urinary excretion. The ranking ability of the Flower-FFQ was assessed by dividing the intake of nutrients and foods as assessed by Flower-FFQ and regular-FFQ over quartiles after which we examined whether persons were ranked into the same, adjacent or extreme quartile. If ≥ 50 % of the participants were classified in the same quartile, this was considered a good outcome^(11)^. Additionally, Pearson and Spearman rank correlations were calculated and classified according to the cut-offs as suggested by Lombard and colleagues, that is, good in case of *r* ≥ 0·50, acceptable in case of *r* 0·20–0·49 and poor in case of *r* < 0·20^(11)^. Nevertheless, given the high probability of correlated errors between the Flower-FFQ and the reference FFQ, we feel that these cut-offs should be interpreted with caution and that correlations should be at least in the upper regions of the acceptable range. All statistical analyses were performed using SAS 9.3.

## Results

Population characteristics of 401 men and women are shown in Table 1. Participants had a mean ± sd age of 54± (11) years, 56 % were ≥ 55 years, 48 % were men and 51 % had a BMI ≥ 25 kg/m^2^. Levels of educational attainment were predominantly medium (31 %) or high (61 %). Participants with a history of myocardial infarction (2 %), stroke (1 %), diabetes (2 %) or cancer (6 %) were rare. Participants completed the Flower-FFQ1, Flower-FFQ2, Flower-FFQ3 and Flower-FFQ4 in ±24, 9, 8 and 9 min (±50 min total), respectively. The regular-FFQ was completed in ±43 min.

**Table 1:** General characteristics of 401 men and women included in the Flower-FFQ validation study

	All		Men (*n* 191)		Women (*n* 210)		Age < 55 years (*n* 175)		Age ≥ 55 years (*n* 226)		BMI < 25 kg/m^2^ (*n* 198)		BMI ≥ 25 kg/m^2^ (*n* 203)	
	*n*	%	*n*	%	*n*	%	*n*	%	*n*	%	*n*	%	*n*	%
Age, years														
Mean	54		57		51		44		62		52		56	
sd	11		10		11		8		4		11		10	
Men	191	48	191	100	0	0	61	35	130	58	71	36	120	59
BMI, kg/m^2^														
Mean	25·6		26·4		24·8		24·9		26·1		22·7		28·3	
sd	3·7		3·3		3·8		3·9		3·4		1·6		2·9	
Waist circumference, cm														
Mean	91		97		85		87		93		82		99	
sd	12		10		10		12		11		8		9	
Education*														
Low	33	8	20	10	13	6	7	4	26	11	9	5	24	12
Medium	125	31	63	33	62	30	51	29	74	33	54	27	71	35
High	242	61	108	57	134	64	116	67	126	56	135	68	107	53
Smoking status*														
Never	184	50	74	41	110	58	107	67	77	36	99	55	85	45
Former	159	43	93	51	66	35	40	25	149	57	71	40	88	46
Current	27	7	14	8	13	7	13	8	14	7	9	5	18	9
Disease history														
Myocardial infarction	7	2	6	3	1	0	0	0	7	3	3	2	4	2
Stroke	3	1	1	1	2	1	1	1	2	1	1	1	2	1
Diabetes mellitus	7	2	6	3	1	0	0	0	7	3	1	1	6	3
Cancer	22	6	14	7	8	4	5	3	17	8	7	4	15	8
Diet during past month	28	7	9	5	19	9	10	6	18	8	11	6	17	8

* Missing values: education 1; smoking 31.

For the Flower-FFQ, the covered level of intake ranged between 93 and 95 % for energy and macronutrients and 93 and 97 % for micronutrients; the covered variance of nutrient intake ranged between 93–97 % and 95–100 %, respectively (Table 2). The covered nutrient intake of the regular-FFQ varied between 94 and 100 % for energy and macronutrients and between 97 and 99 % for micronutrients; the covered variance in nutrient intake ranged between 91–99 % and 62–94 %, respectively.

**Table 2:** Absolute nutrient intakes measured by Flower-FFQ and regular-FFQ with corresponding cross-classification and correlations (*n* 401)

											Flower-FFQ *v*. regular-FFQ				
	Flower-FFQ				Regular-FFQ				Group-level bias†		Cross-classification by quartiles			Pearson‡	Spearman‡
	Mean	se	M1	M2	Mean	se	M1*	M2*	%	95 % CI	Same (%)	Adjacent (%)	Extreme (%)	*r*	*r*
Energy, kJ/d	7953	104	93	94	8718	108	97	95	−8·77	−11·85, −5·70	50	39	2	0·68	0·68
Energy, kcal/d	1897	25	93	94	2080	26	97	95	−8·80	−10·31, −7·28	49	40	2	0·68	0·68
Total carbohydrates, en%	43	0·3	93	96	43	0·3	96	93	0·00	−0·13, 0·13	56	41	2	0·69	0·68
Total carbohydrates, g/d	204	2·9	93	96	222	3·0	96	93	−8·11	−8·65, −7·57	50	39	1	0·73	0·71
Mono/disaccharides, g/d	87	1·6	NC	NC	96	1·6	98	93	−9·38	−9·82, −8·93	50	40	2	0·65	0·67
Polysaccharides, g/d	118	1·8	NC	NC	125	2·0	94	89	−5·60	−6·06, −5·14	49	40	1	0·73	0·71
Fibres, g/d	23	0·3	NC	NC	25	0·3	97	99	−8·00	−8·16, −7·84	46	41	2	0·69	0·67
Total protein, en%	16	0·1	93	93	15	0·1	97	91	6·67	6·59, 6·74	44	40	2	0·54	0·57
Total protein, g/d	75	0·9	93	93	76	0·9	97	91	−1·32	−1·60, −1·03	44	44	3	0·62	0·63
Plant-based protein, g/d	32	0·5	93	96	35	0·5	96	91	−8·57	−8·80, −8·34	46	44	1	0·72	0·71
Animal protein, g/d	43	0·7	92	90	41	0·6	98	90	4·88	4·59, 5·16	47	40	4	0·58	0·61
Total fat, en%	35	0·3	94	95	36	0·3	97	93	−2·78	−2·92, −2·64	43	40	2	0·57	0·56
Total fat, g/d	74	1·3	94	95	83	1·3	97	93	−10·84	−11·23, −10·46	48	37	1	0·62	0·64
SFA, g/d	26	0·5	93	94	28	0·4	97	92	−7·14	−7·38, −6·91	50	37	2	0·63	0·65
MUFA, g/d	26	0·5	93	93	30	0·5	97	91	−13·33	−13·58, −13·09	45	41	2	0·58	0·61
PUFA, g/d	15	0·3	95	97	18	0·3	97	93	−16·67	−16·85, −16·48	45	42	3	0·54	0·59
EPA, g/d	0·12	0·01	NC	NC	0·09	0·00	99	84	33·33	33·27, 33·40	50	37	2	0·54	0·63
DHA, g/d	0·16	0·01	NC	NC	0·11	0·00	99	77	45·45	45·40, 45·51	51	35	1	0·56	0·65
Ethanol, en%	3·17	0·17	95	97	4·18	0·22	100	99	−24·16	−24·41, −23·92	63	31	2	0·78	0·78
Ethanol, g/d	8·4	0·45	95	97	12	0·70	100	99	−30·00	−30·42, −29·58	64	30	2	0·77	0·79
Retinol equivalents, µg/d	1127	27	97	100	1367	35	98	94	−17·56	−19·77, −15·35	42	36	4	0·52	0·47
Vitamin B_2_, mg/d	1·35	0·02	94	96	1·50	0·02	97	89	−10·00	−10·04, −9·96	46	39	2	0·54	0·62
Vitamin B_6_, mg/d	1·36	0·02	94	95	1·63	0·02	97	84	−16·56	−16·61, −16·52	42	38	4	0·43	0·46
Folic acid, µg/d	269	4·1	95	96	278	4·1	98	78	−3·24	−3·91, −2·56	47	38	4	0·49	0·58
Vitamin B_12_, µg/d	5·1	0·17	96	98	4·4	0·10	98	89	15·91	15·72, 16·10	43	40	3	0·41	0·56
Vitamin C, mg/d	101	2·1	95	98	89	1·8	97	62	13·48	12·89, 14·07	48	39	2	0·61	0·65
Vitamin E, mg/d	12	0·2	96	98	13	0·2	99	90	−7·69	−7·84, −7·54	39	43	3	0·45	0·51
Ca, mg/d	964	17·4	96	98	971	15·5	98	94	−0·72	−2·18, 0·74	48	38	3	0·53	0·62

NC, not calculated.

* Based on Eussen (2019) paper.

† % Group-level bias = (mean intake Flower-FFQ/mean intake regular-FFQ) × 100–100.

‡ All *P* < 0·0001. M1, covered nutrient intake; M2, covered variance in nutrient intake.

The Flower-FFQ and regular-FFQ showed relatively similar mean intakes for energy and most macronutrients (group-level bias ≤ 10 %) (Table 2). Percentage differences for macronutrient-fractions were somewhat more diverse. Intakes of most micronutrients were rather comparable with a group-level bias < 10 %. Group-level bias was > 10 % for EPA (0·12 *v*. 0·09 g/d), DHA (0·16 *v*. 0·11 g/d) and ethanol (8 *v*. 12 g/d). Although group-level bias was modest for most nutrients, bias generally pointed towards lower nutrient intake estimates as assessed by the Flower-FFQ. Spearman correlations were *r* 0·6–0·8 for all macronutrients and macronutrient-fractions (g/d) and *r* 0·4–0·8 for micronutrients. Moreover, the Flower-FFQ classified ≥ 80 % of the participants in the same or adjacent quartile as the regular-FFQ for all nutrients under study except retinol (78 %). Misclassification ≥ 5 % in the extreme quartile did not occur for any of the nutrients under study.

For food groups, potatoes (71 *v*. 71 g/d), bread (130 *v*. 129 g/d), eggs (13 *v*. 14 g/d), fruit (177 *v*. 190 g/d), cereals (8 *v*. 8 g/d), legumes (13 *v*. 14 g/d), vegetables (172 *v*. 167 g/d), sweets (26 *v*. 29 g/d), tea (284 *v*. 275 g/d), meat (67 *v*. 68 g/d) and fruit juice (47 *v*. 50 g/d) showed the most comparable mean absolute intake estimates for the two FFQ (Table 3). Group-level bias for these food groups ranged from 0 to 10 %. Absolute intake estimates substantially differed between the two FFQ for alcoholic beverages (108 *v*. 167 g/d), soft drinks (27 *v*. 20 g/d), savoury snacks (25 *v*. 35 g/d), nuts/seeds (14 *v*. 20 g/d) and fish (30 *v*. 24 g/d). Spearman correlations ranged from *r* 0·4 to 0·6 (soft drink, vegetables, savoury snack, artificially sweetened beverages, nuts/seeds, cheese, pasta, legumes, rice, soup and fish) to *r* ≥ 0·8 (tea). The Flower-FFQ classified ≥ 80 % of the participants in the same or adjacent quartile as the regular-FFQ for all food groups, except vegetables (79 %).

**Table 3:** Absolute food intakes measured by Flower-FFQ and regular-FFQ with corresponding cross-classification and correlations (*n* 401)

									Flower-FFQ *v*. regular-FFQ				
	Number of food items included		Flower-FFQ		Regular-FFQ		Group-level bias		Cross-classification by quartiles*			Pearson†	Spearman†
	Flower-FFQ	Regular-FFQ	Mean	se	Mean	se	%	95 % CI	Similar (%)	Adjacent (%)	Extreme (%)	*r*	*r*
Potatoes, g/d	8	6	71	2·5	71	2·7	0·0	−0·9, 0·9	44	43	2	0·67	0·62
Alcoholic beverages, g/d	7	6	108	6·3	167	10·8	−35·3	−37·0, −33·7	60	35	2	0·73	0·78
Bread, g/d	11	13	130	2·9	129	3·0	0·8	0·1, 1·5	61	33	1	0·80	0·77
Eggs, g/d	2	2	13	0·6	14	0·5	−7·1	−7·6, −6·7	65	29	6	0·59	0·61
Soft drinks, g/d	2	1	27	3·3	20	2·7	35·0	33·0, 37·0	70	19	11	0·33	0·58
Fruit, g/d	6	7	177	5·7	190	6·0	−6·8	−8·0, −5·7	58	32	2	0·65	0·70
Cake and cookies, g/d	6	5	29	1·2	34	1·2	−14·7	−15·3, −14·2	49	38	2	0·57	0·65
Vegetables, g/d	19	13	172	4·3	167	4·2	3·0	2·1, 3·9	44	35	3	0·55	0·53
Savoury snacks, g/d	4	7	25	1·1	35	1·5	−28·6	−29·1, −28·0	43	41	4	0·52	0·55
Cheese, g/d	6	8	31	1·2	28	1·1	10·7	10·1, 11·3	42	43	2	0·50	0·57
Coffee, g/d	2	1	391	11·2	445	14·3	−12·1	−13·8, −10·5	67	28	5	0·73	0·72
ASB, g/d	1	1	27	3·8	23	3·4	17·4	15·2, 19·6	70	13	17	0·68	0·47
Dairy, g/d	22	31	281	10·1	301	9·1	−6·6	−8·2, −5·1	50	40	1	0·57	0·69
Nuts and seeds, g/d	3	7	14	0·8	20	1·0	−30·0	−30·5, −29·5	44	40	3	0·58	0·57
Cereals, g/d	1	4	8	0·6	8	0·6	0·00	−0·6, 0·6	71	20	9	0·68	0·70
Pasta, g/d	2	2	23	0·9	26	1·0	−11·5	−12·0, −11·0	43	37	4	0·51	0·49
Legumes, g/d	1	1	13	0·9	14	1·0	−7·1	−7·8, −6·5	46	34	6	0·63	0·50
Rice, g/d	2	2	27	1·3	31	1·5	−12·9	−13·6, −12·2	47	36	4	0·50	0·55
Soup, g/d	2	2	49	3·3	44	2·9	11·4	10·0, 12·7	49	36	5	0·58	0·52
Soya foods, g/d	6	7	17	2·8	11	1·6	54·6	52·5, 56·6	79	13	8	0·63	0·69
Sweets, g/d	10	10	26	1·1	29	1·1	−10·3	−10·9, −9·8	54	38	1	0·66	0·74
Tea, g/d	5	3	284	12·4	275	12·8	3·3	1·2, 5·4	59	36	1	0·82	0·83
Fats, oils and sauces, g/d	32	38	38	1·1	43	0·9	−11·6	−12·0, −11·2	47	42	3	0·61	0·66
Fish, g/d	11	11	30	1·4	24	0·8	25·0	24·3, 25·7	45	36	2	0·44	0·53
Meat, g/d	24	19	67	1·9	68	1·9	−1·5	−2·1, −0·8	54	34	2	0·71	0·69
Fruit juice, g/d	4	2	47	3·8	50	3·5	−6·0	−7·4, −4·6	54	33	3	0·61	0·65
Water, g/d	1	1	383	15·9	17	2·7	2153	2143, 2163				0·01	0·07

ASB, artificially sweetened beverages.

* Eggs, coffee and cereals were analysed by tertiles due to their distribution; despite the distribution of ASB, soft drink and soya data, these groups were analysed by quartiles, which resulted in three relatively equal groups for both FFQ; water was not analysed due to large questionnaire differences.

† All, except water, *P* < 0·0001.

**Fig. 3:**
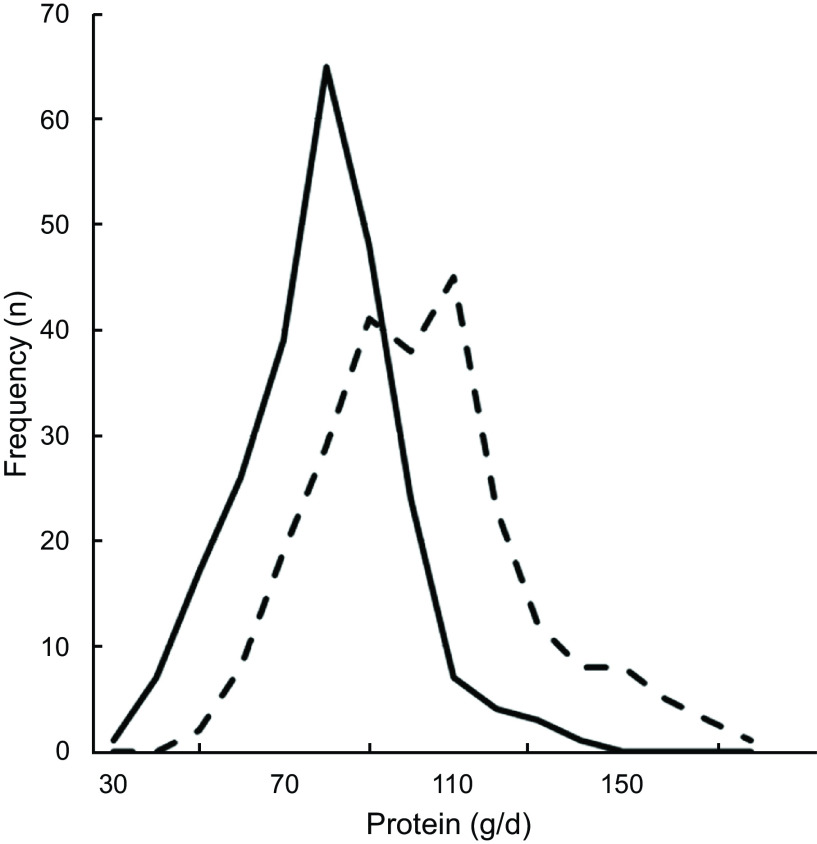
Estimated protein intake distribution based on the Flower-FFQ and urinary excretion. 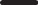, protein intake from Flower-FFQ (g/d); 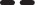, protein intake based on excretion (g/d)

**Fig. 4:**
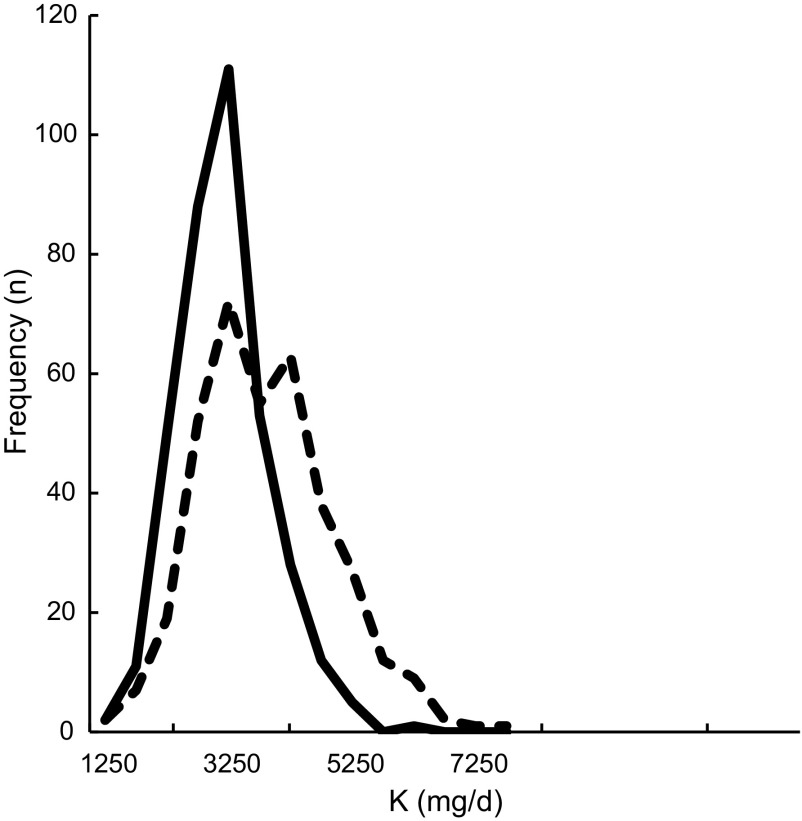
Estimated K intake distribution based on the Flower-FFQ and urinary excretion. 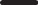, K intake from Flower-FFQ (mg/d); 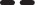, K intake based on excretion (mg/d)

Comparing the Flower-FFQ data on total protein intake (74 se 1·2) with the mean urinary N excretion (98 se 1·6) showed a ˜24 % underestimation of protein intake, which is visually displayed in Fig. 3 (*n* 242). In addition, 75 % of the participants were classified in the same or adjacent quartile when comparing the FFQ and urine data; corresponding Pearson and Spearman correlations were 0·41 and 0·40. The mean self-reported K intake was 3169 mg (se 39), whereas the urinary K excretion was quantified at 3878 (se 64) mg, indicating an 18 % underestimation by the Flower-FFQ (*n* 361). The Flower-FFQ and urinary data classified 73 % of the participants in the same or adjacent quartile and 3 % in the extreme quartiles; Pearson and Spearman correlations between the two methods were *r* 0·33 and *r* 0·37 (Fig. 4).

## Discussion

We developed a new type of FFQ consisting of four short FFQ that can be administered at different time points. This FFQ is assumed to be less burdensome than a regular long FFQ and therefore expected to be less sensitive to measurement error. No usability testing was performed, but the online system registered a ± 7 min longer completion time for the whole Flower-FFQ compared with the regular-FFQ. Regarding the nutrient and food intake estimates, the Flower-FFQ yielded somewhat lower intake estimates than the validated regular-FFQ. Most importantly, as illustrated by correlations ≥ 0·40 and a ranking agreement ≥ 80 % (i.e., ranking in the same or adjacent quartile as the regular-FFQ), the ranking ability of the Flower-FFQ was promising for most nutrients and foods.

Before elaborating on the results of this validation study, several methodological issues warrant attention. First, a validated regular-FFQ was used as the reference method to evaluate the Flower-FFQ. As both FFQ rely on memory, same food composition tables and similar measures to estimate portion sizes, the true performance of the Flower-FFQ may represent an overestimation due to correlated errors. Repeated measures of biomarkers, 24-h dietary recalls or diet records share less correlated errors with the questionnaire under study and would have been more suitable reference methods^(6,25)^. Nevertheless, the regular-FFQ has been shown to have a good ranking ability (*r* 0·82) with respect to the estimated energy intake as compared with the actual energy intake (i.e., based on provided foods and reported free-food items) among 516 men and women participating in controlled dietary intervention studies^(14)^. Moreover, an acceptable to good ranking ability has been observed for a broad variety of nutrients and food groups using multiple 24-h recalls (*n* 128)^(15)^. Finally, Feunekes and colleagues showed strong Pearson correlations for total fat (*r* 0·78) and saturated fat intakes (*r* 0·75) as measured by the regular-FFQ and dietary history; correlations between adipose tissue fatty acids and regular-FFQ were *r* 0·57 for linoleic acid and *r* 0·52 for PUFA in participants with a stable body weight^(20)^. Given these previous validation results of the regular-FFQ as well as the fact that we did assess actual validity by means of urinary N and K, we feel that the current validation study provides solid background data on the performance of the Flower-FFQ. The second methodological issue that needs to be mentioned is that the Flower-FFQ was administrated over a 2-year period. Although partial correlations between dietary variables adjusted for assessment date of the FFQ did not differ from unadjusted correlations, we can merely speculate about potential time effects of the order of administration of the different FFQ in relation to the collected dietary data. However, given the fact that the inclusion of participants was spread between June 2011 and February 2013, where the FFQ were more or less randomly distributed over the year, we do not assume major time effects. Third, our analyses were conducted using data of a subsample (*n* 401) of the total study population (*n* 2048), which comprises slightly older participants (54 *v*. 51 years) and a lower proportion of men (48 % *v*. 52 %). Fourth, one must also bear in mind that our population is higher educated than the general Dutch population (12) and that previous analyses using the National Dietary Assessment Reference Database database have shown higher attenuation factors among those with a higher educational attainment^(26)^. Thus, validity measures of the Flower-FFQ may be lower in populations with a lower educational attainment. Although our sample was mainly high educated (higher secondary education, higher vocational education or university, 61 %) or medium (lower secondary or intermediate vocational education, 31 %) educated, comparing Spearman correlations for these two groups indeed showed substantial differences (i.e., *r* > 0·10 difference) for some of the key nutrients and food groups under study, particularly for total fat, cheese, fish and meat. Further analyses within a more diverse population with respect to educational attainment are needed to draw more definite conclusions on this aspect. Finally, a strength of the current study is that we used multiple statistical approaches to assess the validity of the FFQ, which has been suggested to be most optimal to assess the robustness of the validation process^(6,11)^.

Our results for macronutrient(-fractions) (g/d) showed high group-level bias for EPA, DHA and ethanol. DNFCS data show median EPA/DHA intakes of Dutch men and women around 0·10 and 0·09 g/d, which is in line with the estimates resulting from the regular-FFQ^(27)^. The difference in EPA and DHA intake between the two FFQ was also reflected by a difference in fish intake. As both FFQ included eleven highly comparable food items to assess the intakes of EPA and DHA, absolute intake differences are unlikely to be explained by differences in design between FFQ. However, the timing of the two methods did not fully overlap and may therefore account for some of the difference between the methods (including possible seasonal variation), that is, the Flower-FFQ assessing fish intake (FFQ2) was administered between August 2012 and March 2015, whereas the regular-FFQ was completed between December 2011 and August 2014. For ethanol, the Flower-FFQ yielded lower intakes than the regular-FFQ; this difference was also reflected in a lower intake of alcoholic beverages. DNFCS data showed median ethanol intakes of 16·1 and 3·7 g/d for men and women^(27)^, which was 8·9 and 4·6 g/d in our sample. Again, the number of items assessing ethanol intake of the regular-FFQ and Flower-FFQ was rather similar. However, the timing of both methods did not fully overlap, the regular-FFQ had a slightly higher covered (variance in) nutrient intake than the Flower-FFQ and the question structure of the FFQ somewhat differed. With respect to the latter, the regular-FFQ quantified the consumption frequency of each type of alcoholic beverage separately, whereas the Flower-FFQ first quantified the consumption frequency of the total number of alcoholic beverages consumed and thereafter identified the type of alcoholic beverage consumed. Particularly this questionnaire structure may account for some of the observed differences between the two questionnaires, which has been illustrated by a previous review on alcohol intake assessment. Specifically, directly assessing consumption frequency of specific alcoholic beverages resulted in 19 % higher alcohol intake estimates compared with a situation in which first the total number of alcoholic beverages was assessed followed by a more detailed assessment of the specific types of beverages consumed^(28)^. Nevertheless, despite these differences in – rather low – absolute EPA, DHA and ethanol intake levels, these data still provide valuable information for epidemiological purposes. Namely, in nutritional epidemiology, the ranking of participants according to their intake levels is usually more relevant than absolute intakes. Relating to this ranking ability, correlations and cross-classification of the Flower-FFQ with the regular-FFQ showed good results. Correlations for most macronutrients and macronutrient-fractions in g/d were *r* 0·6–0·8 and 85 % up to 94 % of the population ranked in the same or adjacent quartile as compared with the regular-FFQ. Moreover, for the specific nutrients with a relatively high rate of misclassification in absolute intakes, correlations and cross-classification results were *r* 0·6–0·8 (i.e., EPA, DHA, ethanol) as well. Thus, despite absolute misclassification, such variables can be confidently used for epidemiological purposes. This is further accentuated by the fact that the validity measures of the Flower-FFQ are comparable to the results of previous studies exploring the validity of Dutch FFQ^(5,26,29,30)^. To illustrate, compared with the FFQ-NL1.0, the Flower-FFQ showed comparable or higher correlations for energy (*r* 0·68 *v*. *r* 0·43), macronutrients (protein *r* 0·63 *v*. *r* 0·38, carbohydrates *r* 0·71 *v*. *r* 0·54, fat *r* 0·64 *v*. *r* 0·30), ethanol (*r* 0·79 *v*. *r* 0·77) as well as EPA (*r* 0·63 *v*. *r* 0·33) and DHA (*r* 0·65 *v*. *r* 0·28). Cross-classification results for both FFQ were rather comparable as well^(26)^. However, we do need to indicate that the FFQ-NL1.0 was validated against multiple 24-h recalls, whereas we used an FFQ as the reference method. Due to more correlated errors with the reference method, our validity measures are therefore probably inflated.

As can be expected, the absolute intake differences for micronutrients were larger and more diverse than for macronutrients. Group-level bias percentages ranged from –17·6 % for retinol equivalents to 15·9 % for vitamin B_12_, showing lower Flower-FFQ estimates for retinol, vitamin B_2_, vitamin B_6_ and folic acid and vitamin E, and higher estimates for vitamin B_12_ and vitamin C. However, with correlations and cross-classification results for micronutrients ranging from *r* 0·47 (78 % in same or adjacent quartile) for retinol to *r* 0·65 for vitamin C (87 % in same or adjacent quartile) and *r* 0·62 (86 % in same or adjacent quartile) for Ca, results are still well within the range as suggested by Willet and colleagues (*r* 0·4–0·7)^(31)^. Moreover, also for the micronutrients our validity measures are generally in line with previous validation studies of Dutch FFQ, for instance when comparing the Flower-FFQ with the FFQ-NL1·0, vitamin B_6_ showed correlations of *r* 0·46 *v*. *r* 0·28, folic acid of *r* 0·58 *v*. *r* 0·30, vitamin B_12_ of *r* 0·56 *v*. *r* 0·28 and Ca of *r* 0·62 *v*. *r* 0·42^(26)^.

Our results on food groups are also fairly comparable to preceding FFQ validation studies^(15,26)^. The most notable results are those for water and soft drinks. The explanation for the extreme misclassification rate of water is clear. Whereas the regular-FFQ only assesses bottled water, the Flower-FFQ assesses both tap and bottled water. The discrepancy for soft drinks may be explained by the fact that the Flower-FFQ queries for both regular soft drinks and energy drinks, whereas the regular-FFQ only queries for regular soft drink. As for nutrients, cross-classification and correlations of all other food groups were generally very acceptable with about half of the food groups showing moderately strong associations, and about half of the food groups showing moderate correlations. In line, cross-classification results were generally good where only vegetables showed a comparability below 80 % in the same or adjacent quartile (i.e., 79 %).

In this validation study, we also had the opportunity to compare the intakes of protein and K to their level of urinary excretion, which indicated a ˜24 and 18 % underestimation in protein and K intake. For protein, our results are within the range of results as observed in a pooled analyses of five studies by Freedman and colleagues showing a 10–29 % underestimation for protein^(32)^. For K, the Flower-FFQ showed a substantially higher underestimation than the 5–6 % observed in a pooled analyses by Freedman and colleagues^(33)^. The timing of the urine sampling *v*. the assessment period of the FFQ did not fully overlap and we only collected a single urine sample, which may explain the higher rates of underestimation as compared with the previous validation studies. Moreover, it needs to be mentioned that the Flower-FFQ was not specifically developed to assess K. Nevertheless, for both nutrients the ranking ability was acceptable, that is, 75 % (*r* 0·41) and 74 % (*r* 0·33) of the participants were classified in the same or adjacent quartile.

In conclusion, although group-level bias was relatively high for some nutrients, all nutrients and foods showed a good ranking ability, which suggests that the Flower-FFQ is a suitable tool to study a wide variety of diet–disease associations.
